# *In vivo* evaluation of the antibacterial properties of a poly-*ε*-lysine and hyaluronic acid coated intramedullary implant in a New Zealand White rabbit model

**DOI:** 10.1371/journal.pone.0343597

**Published:** 2026-03-04

**Authors:** Julia L. van Agtmaal, Sanne W. G. van Hoogstraten, Noémie Reinert, Cynthia Calligaro, Rajendra Kasinath, Claudia Zindl, Stephan Zeiter, Nihal Engin Vrana, Tim J. M. Welting, Jacobus J. C. Arts

**Affiliations:** 1 Department of Orthopaedic Surgery, Research School CAPHRI, Maastricht, the Netherlands; 2 AO Research Institute Davos, AO Foundation, Davos, Switzerland; 3 SPARTHA Medical, Strasbourg, France; 4 DePuy Synthes Biomaterials, Warsaw, Indiana, United States of America; 5 Orthopaedic Biomechanics, Department of Biomedical Engineering, Eindhoven University of Technology, Eindhoven, the Netherlands; Hamadan University of Medical Sciences, IRAN, ISLAMIC REPUBLIC OF

## Abstract

Peri-prosthetic joint infection (PJI) is a severe complication that can arise following joint replacement surgery. PJI treatment is extremely complex due to the formation of bacterial biofilms and the emergence of antimicrobial resistance (AMR) to antibiotic classes commonly used in the treatment of PJI. This critical development highlights the urgent need for novel prophylactic strategies that do not rely on conventional antibiotic agents to prevent bacterial adherence and subsequent biofilm formation on implant surfaces. This study evaluated the contact-killing properties of a supramolecular poly-epsilon-lysine and hyaluronic acid (PEL-10/HA-1) coating on an intramedullary nail in a New Zealand White (NZW) rabbit model. Fifteen female NZW rabbits were inoculated with *Staphylococcus aureus* (JAR 060131, 5.9 x 10^4^ CFU in 100 µL PBS) in the right humerus. Seven received an uncoated nail, while eight received a PEL-10/HA-1 coated titanium alloy nail. After 7 days, the rabbits were euthanized for microbiological analysis of the nail and surrounding tissues. Remarkably, microbiological analysis showed that 4/8 of the rabbits with a coated nail had 0 CFU on the nail, versus 1/8 of the rabbits with an uncoated nail. The rinsing solution, soft tissue, and bone samples from the rabbits with a coated nail were more often culture-negative than the samples from rabbits with an uncoated nail. However, no statistically significant differences were observed between the CFUs of the coated and uncoated groups. There were no statistically significant differences between the coated and uncoated groups in other infection indicators, including white blood cell count, C-reactive protein, and plasma protein electrophoresis. While the PEL-10/HA-1 coating had a bacteriostatic and bactericidal effect *in vitro*, this effect did not translate to *in vivo*, highlighting a translational gap. The PEL-10/HA-1 coating must be optimized to enhance its antimicrobial effect, ensuring the same promising in vivo effect as previously observed *in vitro.*

## Introduction

Peri-prosthetic joint infection (PJI) is a severe complication that can arise following primary and revision joint replacement surgery, affecting implant survival, as well as the patient’s functional outcomes and quality of life [1,2]. PJI poses a substantial burden on healthcare systems, as it is one of the leading causes of implant revision surgeries and often requires a complex treatment strategy and prolonged hospitalization [3,4]. The primary pathogens responsible for PJI are the gram-positive *Staphylococcus aureus* (*S. aureus*) and *Staphylococcus epidermidis*, and the gram-negative *Escherichia coli* (*E. coli*) and *Pseudomonas aeruginosa*, which can form a biofilm on implant surfaces [5,6]. Biofilms are complex communities of bacteria embedded in a self-produced extracellular matrix, acting as a protective barrier against the host immune response and antibiotic treatment [7]. Biofilm formation is a major cause of chronic infections, as bacteria within the biofilm can exhibit low metabolic activity, making them less susceptible to antibiotics and the immune system, contributing to persistent and difficult-to-treat infections [8]. Studies have shown a fivefold higher mortality rate for patients with PJI compared to those who had undergone an uninfected joint replacement, underscoring the severity of these infections [9,10]. The emergence of antimicrobial resistance (AMR) to antibiotic classes commonly used in PJI further complicates PJI treatment [11]. AMR poses a global health threat, as a recent study estimated that there will be 1.91 million annual deaths directly attributable to AMR in 2050 [12]. This alarming trend underscores the urgent need for novel treatment strategies that do not rely solely on antibiotics to prevent biofilm formation on implant surfaces.

A novel polyelectrolyte-based supramolecular antimicrobial contact-killing coating has been developed to prevent bacterial adhesion. Poly-epsilon-lysine (PEL-10), a polycation, has shown strong antimicrobial properties by disrupting bacterial cell membrane peptidoglycans and lipopolysaccharides, and hyaluronic acid (HA-1), a biocompatible polysaccharide, has antifouling properties that reduce bacterial attachment and promote tissue integration [13,14]. Together, these materials can form a thin coating that can potentially prevent bacterial colonization on implant surfaces. Previous studies have shown significant antibacterial properties, up to a 5-log reduction in colony-forming units (CFU) against *S. aureus* and *E. coli*, and biocompatibility of the PEL/HA coating *in vitro* [15]*.* An important next step toward the clinical application of the coating is to assess its antibacterial efficacy in an *in vivo* setting. This study aimed to determine the contact-killing properties of the coating on an intramedullary (IM) nail in an *in vivo* New Zealand White (NZW) rabbit model, a well-established preclinical model for studying infection prevention in orthopedic surgery [16–19]. The PEL-10/HA-1 coating has strong contact-killing properties *in vitro*, so it is expected to prevent bacterial adherence to the implant *in vivo* [15]*.* This study aimed to assess the potential of this novel contact-killing PEL-10/HA-1 coating to reduce bacterial adhesion and thereby biofilm formation on orthopedic implants.

## Methods

### Institutional animal care and ethical approval

Fifteen healthy (based on clinical examination, hematocrit (Hct), and white blood cell count (WBC)) female New Zealand White (NZW) rabbits (Charles River, Sulzfeld, Germany), at the age of 22–36 weeks, with a weight of 3.0–4.5 kg, were included in the study. As treatment success in PJI is not known to differ between the sexes, only female rabbits were chosen, as they are known to fight less than males [20–22]. The rabbits were specific, opportunistic pathogen-free, and Vendor-Assured Free/Plus (SPOF and VAF/Plus). The Canton Grisons, Switzerland Ethical Committee approved this study (ethical approval number 13/2024). All experiments were conducted in accordance with Switzerland’s animal protection laws and regulations. The rabbits were housed, and the surgeries were performed, in a preclinical facility accredited by the Association for Assessment and Accreditation of Laboratory Animal Care (AAALAC). The rabbits were acclimatized and housed in groups for at least 4 weeks before the surgical procedure. Throughout the experiment, the rabbits had food and water ad libitum. The NZW rabbit model for PJI was performed according to the ARRIVE guidelines, a checklist of recommendations for the complete and transparent reporting of research involving animals, and elaborated checklists [16,23]. The humerus was chosen for implantation because it is exposed to lower forces during weight-bearing, especially compared to bones in the pelvic limbs, which lowers the risk of bone fractures, nail displacement, and discomfort for the animal [16].

### Implants and coating

Medical-grade titanium 7%-aluminum 6%-niobium (TAN, ISO 5832/11) IM nails (54 mm long, 2.5 mm diameter) were manufactured at the AO Research Institute (RISystem AG, Davos, Switzerland). Test nails (n = 8) were coated with PEL-10/HA-1 (SPARTHA Medical, Strasbourg, France). Before coating, the nails were sonicated for 5 min in 70% ethanol and dried at room temperature. PEL solution was prepared at 10 mg/mL (PEL-10), and HA solution was prepared at 1 mg/mL (HA-1) in Tris buffer (20 mM) at pH 7.4. The nails were dipped in the PEL-10 solution, ultrasonicated for 15 sec, rinsed in the Tris buffer, dipped in the HA-1 solution, ultrasonicated again for 15 sec, and rinsed again in the Tris buffer. This process was repeated until 36 bilayers, each consisting of a layer of PEL-10 and a layer of HA-1, were obtained, creating a thin coating (0.5–2.5 µm range) based on the electrostatic interaction between the positively charged PEL-10 and the negatively charged HA-1. Coating adhesion to the nail was confirmed by confocal microscopy (S1 Fig). The nails were dried at room temperature and sterilized with UV light for 30 minutes. Before implantation, each nail was packaged individually and autoclaved at 143°C for 8 minutes.

### Bacterial strain

*S. aureus* JAR 060131 (Swiss Culture Collection CCOS 890, Basel, Switzerland), isolated from a patient with an infected hip prosthesis, was used in this study. A previously determined antibiotic susceptibility profile showed JAR 060131 was susceptible to all antibiotics except penicillin [24]. For each rabbit, an individual inoculum was prepared at a target concentration of 5.9 ± 1.3 x 10^4^ CFU in 100 µL of phosphate-buffered saline (PBS, P4417-50TAB, Sigma-Aldrich), based on previous studies [17–19]. The day before the surgery, JAR 060131 was inoculated from a frozen stock (stored at −80°C) into 5 mL Tryptic Soy Broth (TSB; CM0129B, Thermo Fisher Scientific) and incubated for 12–24 h at 37°C and 100 rpm. On the day of the surgery, 100 µL of the overnight culture was added to 5 mL of pre-warmed TSB and incubated for 2–2.5 h at 37°C and 100 rpm to ensure the bacteria were in their log phase. Two hours before the surgery, the subcultures were centrifuged (7 min, 3220 RCF, RT) and washed twice in PBS. These suspensions were sonicated in a Bandelin Ultrasonic water bath (Model RK 510 H) for 1–3 min, and optical density at 600 nm (OD_600_) was measured using the spectrophotometer (Multiskan GO, Thermo Scientific, SOP PRI045). A final inoculum suspension was prepared by diluting the bacterial culture to an OD_600_ of 0.25, calculated for a 1 mL volume. Bacterial concentrations were quantified by plating serial dilutions of the suspension on blood agar plates (Columbia agar containing 5% defibrinated horse blood, 10025, Liofilchem) and incubating for 24 h at 37°C, after which the CFU could be counted.

### Study design

**Fig 1:**
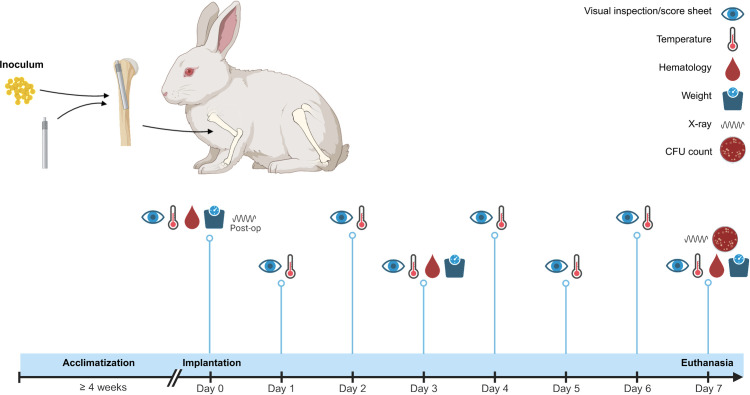
Overview of the study timeline. All rabbits were acclimatized for ≥ 4 weeks, after which they underwent surgery and implantation of a coated or uncoated intramedullary nail in the humerus after inoculation with 5 x 9·10^4^ CFU in 100 µL of phosphate-buffered saline. Visual inspections and temperature measurements were performed daily. Blood was drawn and weight was measured on days 0, 3, and 7. Directly after implantation and on day 7, X-rays were taken. After 7 days, the rabbits were euthanized, and bacterial cultures were made of the nail and surrounding tissues. Created in https://BioRender.com.

A study timeline from surgery to euthanasia is provided in Fig 1. The study population of 15 rabbits all received the bacterial inoculum. Eight rabbits had a TAN-coated intramedullary (IM) nail implanted, with the control group (n = 7) having an uncoated IM nail implanted. The experiment was based on previously published models [17–19,25]. Animals were randomly assigned to a group. All personnel were blinded until all results were analyzed, except for the study director from the animal facility in charge of correct group allocation, and the anesthetist in charge of cross-checking and documenting the allocation of the rabbits.

### Anesthesia and surgical procedure

Each rabbit was weighed and subsequently sedated accordingly, with a combination of medetomidine (Medetor®, Virbac AG, 58407, 0.2 mg/kg IM), midazolam (Dormicum®, Roche, 44448, 0.5 mg/kg IM), and fentanyl (Sintenyl®, Sintetica, 53987, 0.005 mg/kg IM). A blood sample was drawn, and a comprehensive blood count (CBC) was performed as part of the entry test for each rabbit. General anesthesia was induced with propofol to appropriate anesthetic depth (Propofol 1% MCT®, Fresenius, 57029, 0.2 mg/kg IV), rabbits were intubated using a cuffed endotracheal tube with an inner diameter of 3.5 mm (Rüschelit® Super Safety Clear, Ref.: 112482, Rüsch AG, Switzerland) and anesthesia was maintained using Sevoflurane (Sevoflurane Baxter®, Baxter AG, 55999, 1.8–2.2% in 0.6–0.8 L/min oxygen). Rabbits were continuously monitored during surgery using pulse oximetry, capnography, and inspiratory/expiratory anesthesia gas concentration. Carprofen (Rimadyl®, Pfizer AG, 57281, 4 mg/kg IV, 0.4 mL) was administered preoperatively and once daily for 3 consecutive days. Buprenorphine (Bupaq®, Streuli Pharma AG/63081, 0.05 mg/kg IM, 6-8h after surgery) and fentanyl (Fentanyl-Mepha^®^, Matrixpflaster Matrix patches, 12 μg/rabbit for 72 hours) were used as postoperative analgesia. No systemic antibiotics were administered.

**Fig 2:**
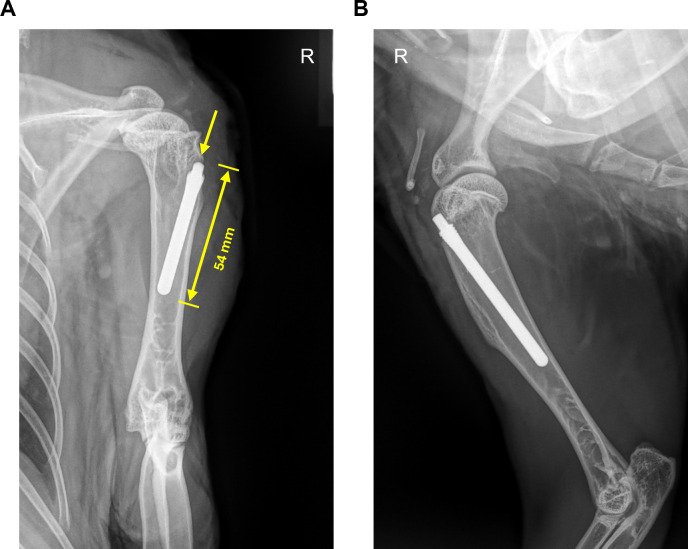
Radiographs of the right humerus taken immediately postoperatively. Craniocaudal **(A)**, with the length of the intramedullary nail, and the insertion point indicated with the yellow arrow, and mediolateral **(B)**.

The rabbits were positioned in left lateral recumbency. The right front limb was clipped, and the skin was aseptically prepared. The surgical area was draped to maintain asepsis of the surgical area. The foot was wrapped with a sterile bandage, and the leg was covered with an iodine adhesive drape (Dermadine Plus®, 20x20cm, Tiaset, San Cipriano, Italy). The surgical steps are presented in S2 Fig. The skin was incised over 1 cm on the lateral aspect of the proximal humerus to expose the insertion of the supraspinatus and infraspinatus tendons. The subcutaneous tissue was sharply dissected along the skin incision, and bipolar cautery was used for hemostasis. The cortex of the proximal humerus was penetrated to enter the medullary cavity just below the growth plate using a ⌀ 2 mm drill bit. Using a 2.5 mm reamer, the medullary cavity was reamed to the length of the intramedullary (IM) nail so that the IM nail could be completely inserted in the medullary canal. Before inoculation and nail insertion, the depth was checked with a test nail, which was subsequently removed. All fluid was removed from the intramedullary canal using suction and an 18G intravenous catheter. The bacterial inoculum (100 µL with 5.9 x 10^4^ CFU) was pipetted into the IM canal, and the nail was inserted. The surgical wound was closed in three layers with absorbable suture material, consisting of myofascial and subcutaneous closure using a simple continuous pattern and skin closure using an intradermal pattern. Orthogonal radiographs of the operated humerus were taken immediately postoperatively (Fig 2) to evaluate implant positioning, and rabbits were monitored in the preparation area until fully awake, before returning them to the animal area.

### Postoperative management

During the first 3 postoperative days, rabbits were housed individually, and if animal compatibility allowed, in pairs for the remainder of the study. Rabbits were checked on the evening of the surgery and twice daily afterward, and scored using a dedicated score sheet, including observations about general demeanor, respiration, inner body temperature, third eyelid protrusion, eating and drinking behavior, defecation, soiling of fur, weight-bearing on the operated limb, paw placement, and inspection of the surgical incision (S1 Table). Bodyweight was recorded 3 and 7 days after surgery. Humane endpoints were defined as a guideline to help with decision-making for premature euthanasia in case of sudden or severe deterioration of the clinical status of the rabbits (S1 Table). Additionally, physiological measurements are needed to assess the health status, as rabbits are prey animals, and they try to hide any discomfort or illness [22]. Therefore, the initial physiological measurements of the rabbits were taken before the surgical procedure, including their weight and blood for hematological analysis. Hematological analysis was performed 3 days and 7 days after surgery, consisting of Hct, WBC, C-reactive protein (CRP), and plasma protein electrophoresis (PPEP) evaluation. Elevated WBC and CRP have been demonstrated to correlate with PJI [16,26,27]. A total rise in proteins in the plasma may indicate inflammation or infection, more specifically, α- and β-globulins levels elevate due to acute inflammation or infection [28,29]. Hct is a standard measurement used to assess anemia, which is associated with chronic disease and may indicate osteomyelitis; however, it is not specific to this condition [28]. Hct results can be found in S1 File. Seven days after surgery, rabbits were euthanized by an intravenous injection of Pentobarbital (Esconarkon®, 300 mg/mL, Streuli Tiergesundheit AG, Uznach, Switzerland). Orthogonal radiographs of the operated humerus were taken to evaluate any change in implant positioning, and a post-mortem macroscopic examination of the external body surface and surgical site was performed.

### Microbiological evaluation

After euthanasia and observation, the rabbits were positioned in left lateral recumbency, and the right front limb was excised at the scapula. The skin was wiped down with 70% ethanol and resected. The skin, radius, ulna, and scapula were resected aseptically, leaving a sterile environment for the humerus and surrounding tissue. Soft tissue was resected until the entry point of the nail was visible. For sample collection, first, soft tissue covering the nail head was resected with a sterile scalpel, and any observed abscesses were collected. This soft tissue was weighed and homogenized (Omni TH, tissue homogenizer TH-02/ TH21649) in 10 mL PBS. Next, the nail was removed from the humerus, rinsed in 15 mL PBS, transferred to a vial with 12 mL PBS, and sonicated for 3 min in a water bath. Last, the humerus was crushed using a sterile Luer bone rongeur, weighed, put into 40 mL PBS, and homogenized (Polytron System PT 3100, Kinematica Ag, Switzerland). All samples were vortexed for 5 seconds. A 200 µL aliquot of the undiluted sample and 10 µL aliquots from each 10-fold serial dilution of the soft tissue and bone homogenate, the rinsing solution, and the nail sonicate were added to blood agar plates. All plates were incubated at 37°C, and bacterial counts were recorded at 24h and 48h. The lower detection limit was 50 CFU/soft tissue, 75 CFU/rinsing solution, 60 CFU/nail, and 200 CFU/bone. For statistical analysis and to visualize the data on a logarithmic scale, 1 CFU per sample was assigned when no growth had occurred. From each rabbit, at least one colony was evaluated by the latex agglutination test (Staphaurex Plus, Oxoid AG) to exclude the growth of other species.

### Statistical analysis

As *in vitro*, a bacterial reduction of 3–5 log was achieved, the expected mean difference (Δ) was estimated at 3 (log reduction), and the standard deviation (σ) for rabbits with a coated nail at 1.5 and for rabbits with an uncoated nail at 2 log, resulting in an effect size (d) of 1.697. With a significance level of α = 0.05 and a power of 0.9, the power calculation resulted in experimental groups of 7. The power calculation was performed using G*Power 3.1.9.7 [30]. Statistical analysis was performed using GraphPad Prism 10.1.2 (GraphPad Software, Inc.) for Windows. The CFU from the coated and uncoated nail groups were compared per sample group with a one-sided unpaired t-test, and data were assumed to be normally distributed. 2-way ANOVA (mixed design with the between-subject factor being the uncoated or coated nail, and the within-subject factor the day of measurement) was used to compare the weight, temperature, and hematology values between the coated and uncoated nails, over time, and between rabbits. P-values <0.05 were considered statistically significant.

## Results

No humane endpoints were reached, and no rabbits were euthanized prematurely; thus, all rabbits were included in the study. The average inoculum of JAR 060131 was 3.76 ± 0.37 x 10^4^ CFU (range 3.17 x 10^4^–4.40 x 10^4^) in 100 µL of PBS per rabbit, with no statistical differences between the two groups (p = 0.4611). All postoperative and post-mortem mediolateral and craniocaudal radiographs showed correct nail placement. In the uncoated group, two rabbits received an additional injection of anti-inflammatory medication on day 4 due to elevated inner body temperature; one also showed increased swelling of the surgical site on day 6. Another rabbit showed increased swelling on days 2 and 3. One rabbit exhibited increased lameness on days 5 and 6, necessitating additional opioid medication, according to the veterinarian’s opinion. In the coated group, two rabbits recovered more slowly than expected from the procedure and received a subcutaneous infusion of 100 mL Ringer’s lactate 1 day postoperatively. Due to continued fighting, two rabbits were separated again and housed individually on day 4.

**Fig 3:**
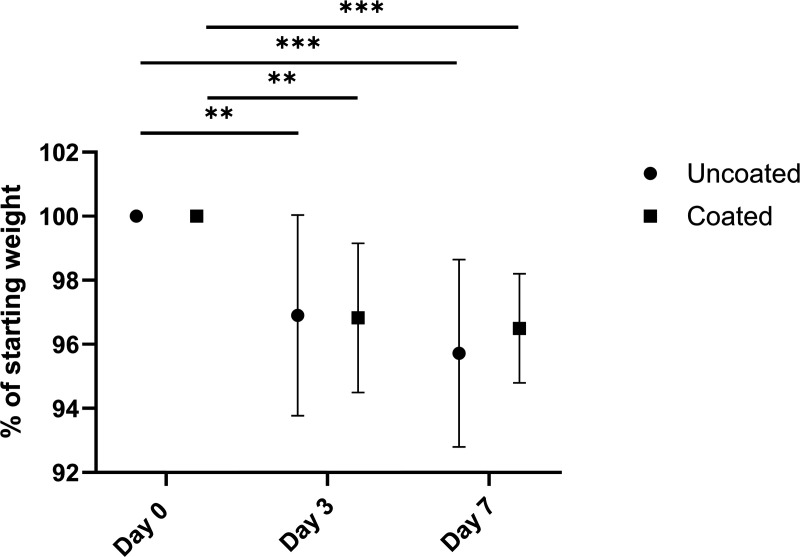
Mean weight change (± standard deviation) throughout the experiment of the rabbits. Presented as a percentage of the starting weight on day 0. The rabbits with uncoated and coated nails are presented in circles and squares, respectively. Statistically significant differences found by multiple comparisons are presented with ** p < 0.01, and *** p < 0.001.

The highest weight loss measured over 7 days was 7.7% for the coated group and 9.1% for the uncoated group, staying sufficiently below the 15% set as a humane endpoint. As shown in Fig 3, the coated group had an average weight reduction of 2.3% from baseline to day 3 and 1.7% from day 3 to day 7, while the uncoated group showed an average weight reduction of 3.1% from baseline to day 3 and 2.9% from day 3 to day 7. There was no statistical difference between the coated and uncoated groups (p = 0.7776); however, a significant difference was observed between the days of measurement (p < 0.0001). Multiple comparisons demonstrated that for the uncoated group, this difference was significant between day 0 and day 3 (p = 0.0060) and between day 0 and day 7 (p = 0.0002). For the coated group, this difference was observed between day 0 and day 3 (p = 0.0025) and between day 0 and day 7 (p = 0.0009). Absolute values of the temperature and weight during the study can be found in S2 and S3 Tables.

### Microbiological analysis

**Fig 4:**
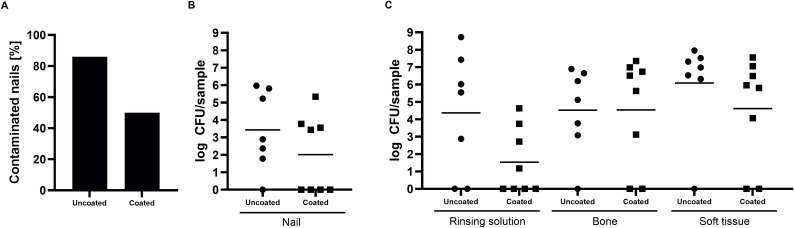
Geometric means of the Staphylococcus aureus colony-forming units (CFU) results. Microbiological evaluation of the implanted nails **(A and B)**, and the rinsing solution, bone, and soft tissue **(C)** after euthanasia of the rabbits on day 7. A one-sided t-test showed no statistical significance between the uncoated and coated groups for all sample groups. To visualize samples with 0 CFU on the log scale, they were changed to 1.

To investigate the antimicrobial effect of the coating, microbiological analysis was performed on the nail, rinsing solution, bone, and soft tissue. Analysis showed no statistical difference in CFU counts between the coated and uncoated groups for all sample materials: nail (p = 0.0663), rinsing solution (p = 0.1373), bone (p = 0.1649), and soft tissue (p = 0.1035) (Fig 4B and 4C). In the coated group, four rabbits completely eradicated the inoculum on the nail and the rinsing solution (Fig 4A). One of these rabbits also eradicated all bacteria in the bone and soft tissue, and two had either 0 CFU in the bone or the soft tissue. However, the coating’s effect appears to be binary, either fully effective or not, as four rabbits demonstrated elevated CFU counts in all materials. One rabbit in the uncoated group only had 60 CFU on the nail and 1.20 ∙ 10^3^ CFU in the bone, which is lower than the starting inoculum, and one rabbit only had bacteria in the soft tissue (2.10 x 10^6^ CFU). The mean differences between the uncoated and coated samples were 1.42 log for the nail, 2.8 log for the rinsing solution, −0.01 log for the bone, and 1.47 log for the soft tissue.

### Hematology

**Fig 5:**
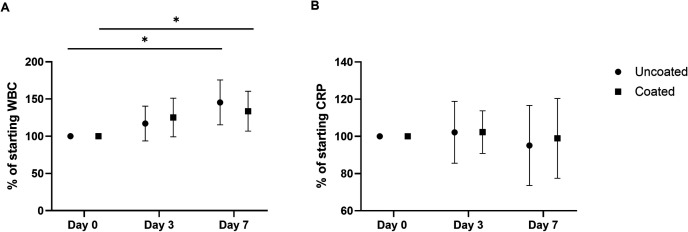
Hematology parameters. A) White blood cell count (WBC), and B) C-reactive protein (CRP) values. The mean values (± standard deviation) throughout the experiment of the rabbits are presented as percentages of the starting values on day 0. The rabbits with uncoated nails are presented in circles, and the coated nails in squares. Statistically significant differences found by multiple comparisons are presented with * p < 0.05.

**Fig 6:**
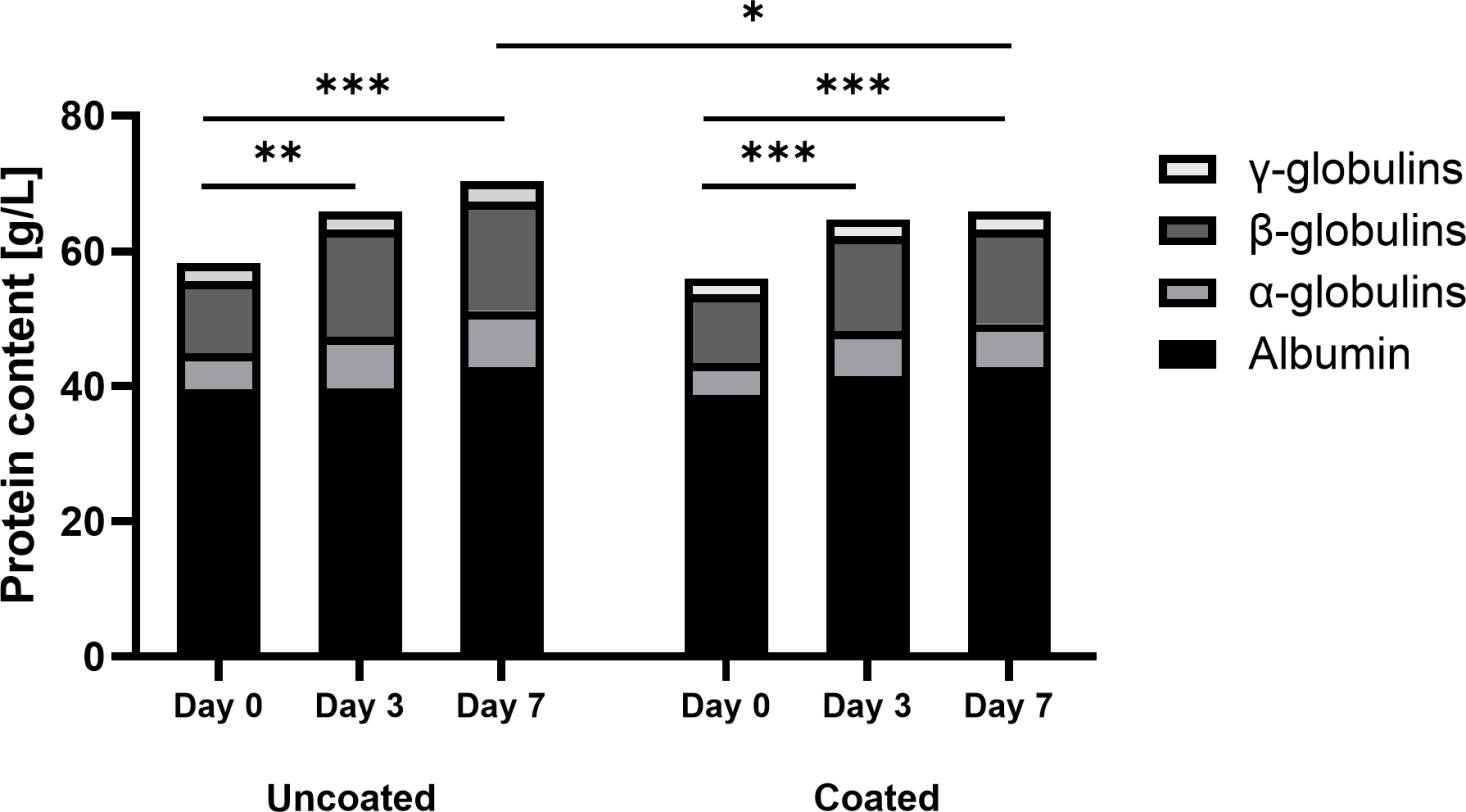
The mean values of the rabbits’ absolute total plasma protein electrophoresis values throughout the experiment. Statistically significant differences found by multiple comparisons are presented with * p < 0.05, ** p < 0.05, and *** P < 0.005.

Hematology parameters were calculated as percentages of the baseline values (Fig 5). In the uncoated group, one rabbit decreased to below the WBC reference range (WBC = 5.5–12.5·10^3^) on day 3 but returned to normal levels on day 7. Moreover, one rabbit had an elevated WBC on day 7. No significant difference was found between the uncoated and coated groups for the WBC (p = 0.8624) and CRP (p = 0.8120). WBC only showed significant differences over time (p = 0.0002). Multiple comparisons demonstrated that this was due to an increase in WBC between days 0 and 7 for both implant groups (uncoated, p = 0.0167; coated, p = 0.0222). CRP showed no significant differences over the days. All absolute values can be found in S4 and S5 Tables. The PPEP evaluation showed a statistically significant difference in total proteins between the coated and uncoated groups (p = 0.0422) and over the days (p < 0.0001) (Fig 6). This effect is most prominent on day 7 (p = 0.0140). This was mainly caused by a significant increase in α- and β-globulins. The level of α-globulins was significantly higher (p = 0.0186) in the uncoated group compared to the coated group and increased significantly over time (p < 0.0001). The main effect between the uncoated and coated groups was on day 7 (p = 0.0137). The level of β-globulins was significantly higher (p = 0.0424) for the uncoated group compared to the coated group, and over the days (p < 0.0001). The albumin level only increased significantly over the days (p = 0.0036), but not between the two groups. The γ-globulins showed no significant difference between the groups or over the days. P-values of the multiple comparisons over the separate days for all measured proteins are presented in Table 1. All absolute values can be found in S5 Table. When the values for all proteins measured were presented as percentages of the baseline value, there was no longer a statistically significant effect between the two groups.

**Table 1:** P-values of the multiple comparisons for the absolute total plasma protein electrophoresis, split per day and group. Ns = not significant (p > 0.05). P-values between groups are in the text.

Day	Albumin		α-globulins		β-globulins		γ-globulins		Total	
	Uncoated	Coated	Uncoated	Coated	Uncoated	Coated	Uncoated	Coated	Uncoated	Coated
0 vs. 3	ns	ns	0.0072	0.0208	0.0008	0.0068	ns	ns	0.0003	<0.0001
0 vs. 7	ns	0.0401	0.0203	0.0014	0.0276	0.0007	ns	ns	<0.0001	<0.0001
3 vs. 7	ns	ns	ns	ns	ns	ns	ns	ns	0.0338	0.7808

## Discussion

PJI following primary and revision joint replacement surgery is a severe complication, affecting orthopedic implant survival, functional outcomes, and quality of life [1,2]. With the rise of AMR, the risk of therapeutic insufficiency for PJI is increasing, as many antibiotics are no longer effective in eradicating infections. Novel treatment strategies bypassing classical antibiotics are required to prevent infection and biofilm formation on the implant surface [11]. This study tested a novel PEL-10/HA-1 coating in an established NZW rabbit model for PJI. The effectiveness of a coating in reducing bacterial adhesion on implant surfaces was determined. While there were no statistically significant differences in CFU between coated and uncoated groups, a higher percentage of rabbits with coated nails had negative cultures compared to those with uncoated nails. Despite the lack of statistical significance, it was observed that no viable bacteria were recovered from four out of eight coated implants. This observed pattern suggests potential clinical relevance, warranting further investigation in larger-scale models.

Given that the PEL-10/HA-1 coating has contact-killing properties, the coating is expected to show the greatest effect on the CFU found on the nail. Four rabbits with coated nails had 0 CFU on the nail and in the rinsing solution, indicating a bactericidal effect. At the same time, four rabbits with coated nails exhibited CFU numbers near the inoculation level. This resulted in a 1.42 log mean reduction for the coated nail compared to the uncoated one, which is regarded as not clinically relevant [31]. Moreover, these results contrast with a previous *in vitro* study, demonstrating up to a 5-log CFU reduction of adherent *S. aureus* for PEL-10/HA-1 coated titanium samples compared to uncoated samples [15]*.* Though 50% of the coated nails remained uncontaminated, potential mechanisms for failure, such as coating degradation by serum proteins or mechanical wear, in the other cases need to be analyzed. The rinsing solution demonstrated larger differences (2.84 log) between the coated and uncoated groups. However, there was a high variation in both groups. No significant reduction was found in the CFU for the bone and soft tissue samples of the coated group compared to the uncoated group, potentially attributable to the lack of direct contact of bacteria colonizing these tissues with the coating. As the implant is near bacteria that persist in the bone and soft tissue, it is at risk of being recolonized by proliferating bacteria. Based on the current results, it cannot be concluded that the PEL-10/HA-1 coating can prevent bacterial adhesion to the implant. However, as half of the rabbits with a coated implant already showed no bacterial adhesion, this study shows the coating’s potential when further optimized.

There were no significant differences between the coated and uncoated groups in other infection indicators, including weight loss, WBC, and CRP. The WBC slightly rose over the days in both groups, which may be a stress reaction to the surgery and the inoculum [16,28]. Slight weight loss in both groups after surgery was to be expected due to the opioid medication (Buprenorphine) on the day of surgery and a fentanyl patch for the first three days post-op. Previous studies with an uncontaminated implant showed that weight returned to normal after 5 weeks [32,33]. The significant elevation of total plasma protein levels (mainly due to the α- and β-globulins) in the uncoated group compared to the coated group can be an indication of acute (bacterial) infection [28,29]. However, this elevation was no longer significant when the values were normalized to the baseline value, and should therefore be interpreted with caution. As immunoglobulins (γ-globulins) mainly consist of antibodies, an elevation indicates chronic inflammation [28,29]. Elevated levels of WBC, CRP, and α-, β-, and γ-globulins measure inflammation. The fact that these values are not elevated for the coated group indicates that the PEL-10/HA-1 coating does not induce a statistically significant inflammatory reaction in the rabbit’s body.

Translating and implementing new antimicrobial techniques remains challenging, and discrepancies between *in vitro* and *in vivo* results are commonly observed. A silver multilayer (SML) coating was previously tested *in vitro* utilizing the same ISO 22196, ASTM E2180-18, and JIS Z 2801 test combination as the PEL-10/HA-1 coating, resulting in a > 3 log reduction [34]. However, when tested in a 7-day NZW rabbit model, the SML coating resulted in a decrease of 1.6 log on the nail and 1.6 log in the rinsing solution [35]. Another coating containing the antimicrobial peptide OP-145 eradicated all bacteria *in vitro*; however, in a 28-day NZW rabbit model, some rabbits developed infections exceeding the inoculum level [18]. *In vitro* findings have been shown to translate effectively to *in vivo* for a gentamicin-hydroxyapatite coating [36]. However, *in vitro* antibiotic resistance was observed, and antibiotics such as gentamicin may impair bone ingrowth around the implant [36,37]. It is unknown if these studies combined coatings with systemic antibiotics. *In vitro* test methods often lack the complexity of *in vivo* systems, and bacterial strains can react differently *in vitro* and *in vivo* [38,39].

Translating *in vitro* results to *in vivo* outcomes is difficult due to non-standardized protocols, weak correlation with clinical results, and the limited predictive reliability of current models [38]. *In vitro* test methods lack complexity and clinical relevance, but *in vivo* situations also vary between animals, and bacterial strains can respond differently *in vitro* and *in vivo* [38,39]. Several often overlooked factors *in vitro* include fluid flow [38], the impact of the immune response [40], the effect of bone marrow and blood serum proteins [41], synovial fluid [42], quorum sensing [43], and the force applied during arthroplasty surgeries [44]. Preclinical *in vivo* models are essential for studying host response, implant integration, and pathogen interaction to help bridge the translational gap to clinical applications [39]. To enhance clinical translation, *in vivo* studies should tailor their methodology and outcome parameters based on the intended use and mechanism of action of the antibacterial method [16]. Additionally, when performing a power calculation, this translational gap from *in vitro* to *in vivo* must be considered, as assuming a 3-log reduction could have resulted in an underpowered calculation for this study.

The choice of a 7-day PJI NZW rabbit model was based on previous studies, ensuring a reliable and standardized framework, suited for testing a contact-killing coating. As *S. aureus* is most prevalent in PJI in countries of the European Union, this study used a clinical methicillin-susceptible *S. aureus* strain [45]. The JAR 060131 *S. aureus* strain has previously been characterized and represents the most prevalent epidemic clones of *S. aureus* [17–19,24,25]. The inoculum levels tested in this study are substantially greater than those typically encountered in the clinical setting, creating a challenge for a coating designed for prevention rather than treatment of PJI [46]. Previous studies using the JAR 060131 strain demonstrated that the inoculum used in this study should be sufficient to create an infection in all untreated control rabbits, yet low enough to avoid inducing systemic sepsis [17–19,25,47]. However, comparable to this study, variation in the control groups in these previous studies was also high. Furthermore, how much of the bacterial suspension came into direct contact with the contact-killing coating is uncertain. Moreover, bacteria might colonize and persist better in peri-implant tissue compared to the implant surface [17,48,49]. Clinically, orthopedic infections usually manifest at lower doses than those inoculated in the rabbit model and are less localized, which cannot be modeled *in vivo* [50–52]. Although the used method of inoculation is widely applied in preclinical PJI research, it has inherent limitations in fully replicating the clinical situation, and alternative approaches (such as pre-infecting implants prior to implantation) likewise face translational challenges and introduce different sources of experimental bias [16].

Moving forward, the *in vivo* on-or-off bactericidal effect of the PEL-10/HA-1 coating needs to be optimized. The PEL-10/HA-1 coating has shown both a bacteriostatic and bactericidal effect *in vitro*. These effects are known to differ based on the concentration of the coating and the test conditions [53]. Further *in vitro* and *in vivo* studies will be needed to evaluate whether coatings with higher PEL-10/HA-1 concentrations improve antimicrobial efficacy while remaining within acceptable biocompatibility thresholds. HA-1 forms a hydrogel structure with a high hydration capacity and hydrophilicity, enhancing its antifouling properties by developing a hydration layer that eliminates binding sites for protein and bacterial adhesion [54,55]. However, the high activity of HA-1 with body fluid proteins can disrupt the hydration layer, reducing the antifouling properties [54,55]. Pre-wetting of the coating before implantation could pre-establish the hydration layer and form a steric barrier, reducing available binding sites for proteins and retaining hydrophilicity [54]. Furthermore, several sterilization procedures should be tested to minimize the chance of potential thermally induced degradation. Since there is currently no comparable alternative commercially available to compare the PEL-10/HA-1 coating, no positive control could be used in this study. Ideally, with the emergence of AMR, alternatives to antibiotics are preferred. However, current practice still relies on combination therapy to prevent systemic infections. Thus, the contact-killing PEL-10/HA-1 coating would likely be used alongside systemic antibiotics to enhance infection control and mitigate orthopedic implant-related infection risks. A future study could therefore include control groups with a coated nail combined with antibiotics and an uncoated nail with antibiotics. For future studies, once the antibacterial efficacy of the coating has been established, osseointegration properties should also be evaluated as an outcome parameter in an *in vivo* study. Although a 7-day study duration is commonly used in NZW rabbits to assess infection progression, extending the duration to up to six weeks [56–61] is preferable in future studies to examine both the long-term antibacterial effect of the coating and its impact on the osseointegration of the implant [16]. In an extended experiment, histopathology can be used to examine in more detail the implant-host interaction, biocompatibility, inflammatory response, and bone ingrowth. Histopathology or microscopy can also be implemented to confirm the presence of a mature biofilm on the control implants.

## Conclusion

This study explored the antimicrobial activity of the PEL-10/HA-1 coating for the first time in an *in vivo* model, for further pre-clinical assessment following promising *in vitro* results. While a bacteriostatic and bactericidal effect was observed for the PEL-10/HA-1 coating *in vitro*, this effect did not fully translate to the *in vivo* situation*,* highlighting a translational gap. Though no significant differences were found between the uncoated and coated groups, samples from the rabbits with a coated nail were more often culture-negative than samples from the rabbits with an uncoated nail. Four out of eight rabbits had an uncontaminated coated nail, indicating a binary effect of the coating. The PEL-10/HA-1 coating will be optimized further by testing if increasing the concentration to PEL-20 and HA-5, or the addition of polyarginine, will enhance its antimicrobial effect while preserving its biocompatibility, ensuring the same promising effect *in vivo* as previously found *in vitro.* The effect on macrophage polarization will also be tested.

## Supporting information

S1 Fig PEL-10/HA-1 coated nails labeled with Poly-L-Lysine coupled with fluorescein isothiocyanate (PLL-FITC) (green), observed with confocal microscopy. No fluorescence is observed under the same conditions for uncoated nails.
(TIF)

S2 Fig Surgical procedure. A) view of the lateral aspect of the proximal humerus after incision of the skin and dissection of the subcutaneous tissue with the tissues retracted using a self-retaining retractor – the insertion of the supraspinatus and infraspinatus tendon are exposed; B) the medullary cavity is reamed; C) fluid is suctioned from the intramedullary canal; D) bacterial inoculation; E) insertion of the intramedullary nail.
(TIF)

S1 File HCT. (DOCX)

S1 Table Score sheet. (DOCX)

S2 Table Weight. (DOCX)

S3 Table Temperature. Values outside the reference range are in bold (38.5–39.5°C).
(DOCX)

S4 Table WBC, HCT, CRP. Values outside the reference ranges are in bold: WBC = 5.5–12.5 [*10^3^/µL]; HCT = 33–50 [%].
(DOCX)

S5 Table Protein electrophoresis. (DOCX)
