# Supplementary figures and images for: In vivo evaluation of the antibacterial properties of a poly-ε-lysine and hyaluronic acid coated intramedullary implant in a New Zealand White rabbit model
Source: PLoS One. 2026 Mar 4;21(3):e0343597. doi: 10.1371/journal.pone.0343597 (PMC12959695; doi:10.1371/journal.pone.0343597)

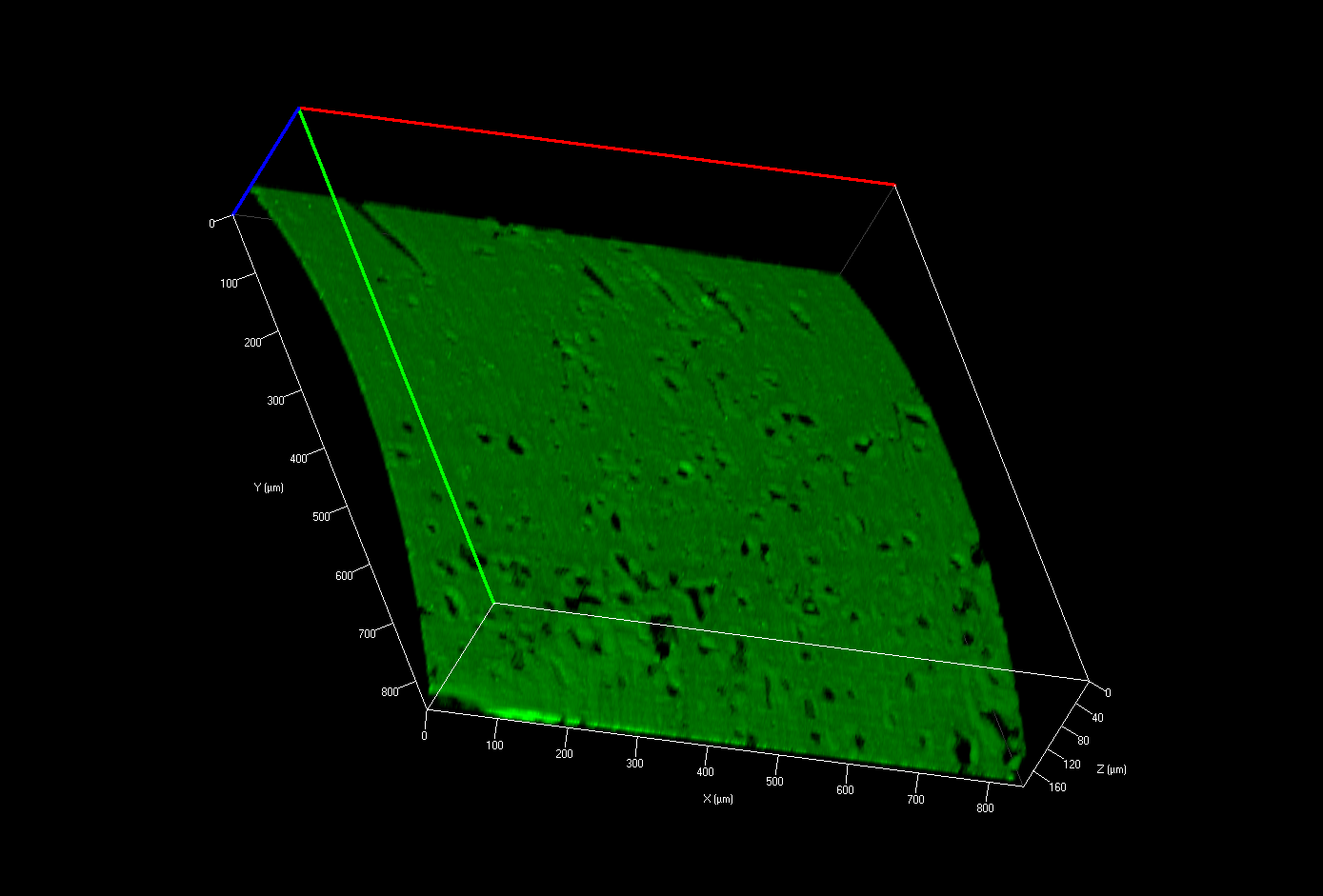

Supplement: S1 Fig — No fluorescence is observed under the same conditions for uncoated nails. (TIF) [file pone.0343597.s001.tif]

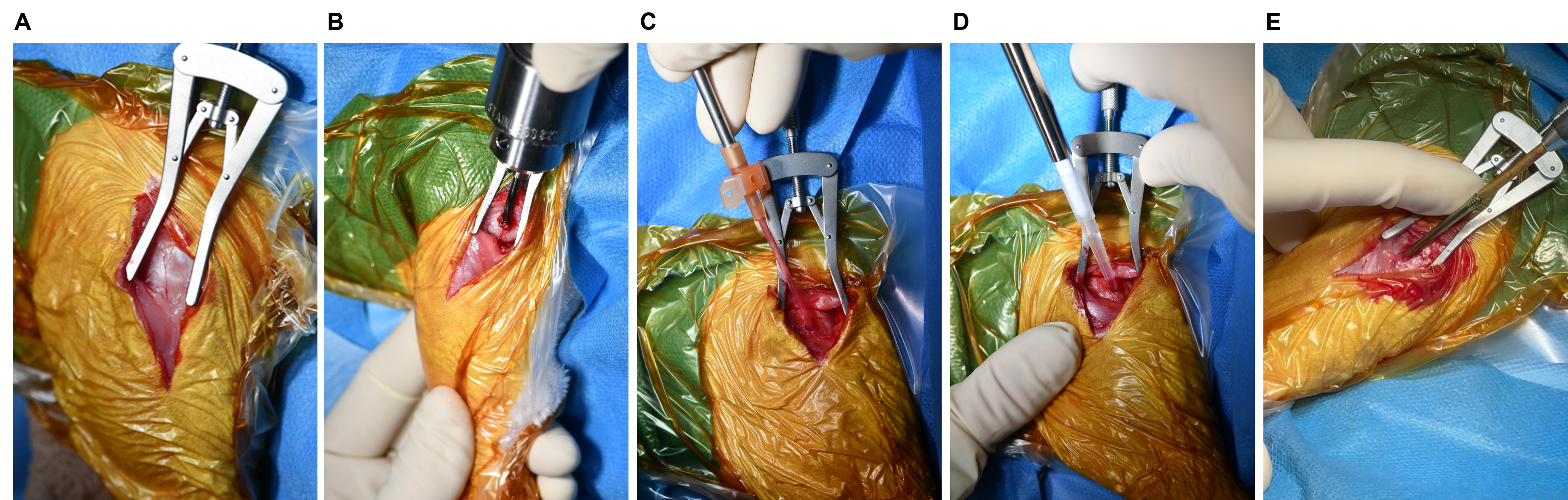

Supplement: S2 Fig — A) view of the lateral aspect of the proximal humerus after incision of the skin and dissection of the subcutaneous tissue with the tissues retracted using a self-retaining retractor – the insertion of the supraspinatus and infraspinatus tendon are exposed; B) the medullary cavity is reamed; C) fluid is suctioned from the intramedullary canal; D) bacterial inoculation; E) insertion of the intramedullary nail. (TIF) [file pone.0343597.s002.tif]
