# Supplementary material for: In vivo evaluation of the antibacterial properties of a poly-ε-lysine and hyaluronic acid coated intramedullary implant in a New Zealand White rabbit model
Source: PLoS One. 2026 Mar 4;21(3):e0343597. doi: 10.1371/journal.pone.0343597 (PMC12959695; doi:10.1371/journal.pone.0343597)
Supplement: S1 File — (DOCX) [file pone.0343597.s003.docx]

**S8. HCT**

The Hct as a percentage of the baseline value is displayed in Supplementary Fig 2. In the uncoated group, one rabbit fell below the Hct reference range (33-50%) on day 3 but returned to normal levels on day 7. Two rabbits were slightly under the Hct reference range on day 7. In the coated group, two rabbits fell slightly under the Hct reference range on day 3 but returned to normal levels on day 7. No significant difference was found between the uncoated and coated groups for the Hct (0.4049). Hct showed no significant differences over the days.

**
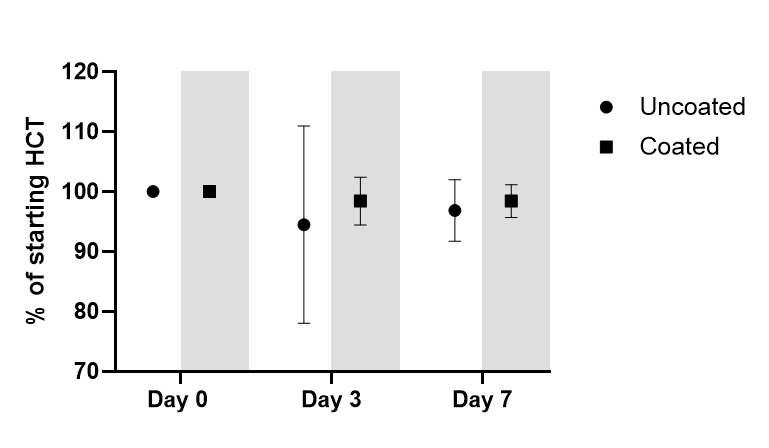
**

**S8 Fig. Hematocrit (HCT) values.** The mean values (± standard deviation) throughout the experiment of the rabbits are presented as percentages of the starting values on day 0. The rabbits with uncoated nails are presented in circles, and the coated nails in squares. Statistically significant differences found by multiple comparisons are presented with * p<0.05.
