# Supplementary material for: In vivo evaluation of the antibacterial properties of a poly-ε-lysine and hyaluronic acid coated intramedullary implant in a New Zealand White rabbit model
Source: PLoS One. 2026 Mar 4;21(3):e0343597. doi: 10.1371/journal.pone.0343597 (PMC12959695; doi:10.1371/journal.pone.0343597)
Supplement: S1 Table — (DOCX) [file pone.0343597.s004.docx]

**S3 Table. Score sheet**

| **Parameter** | **Observation** | **Score** |
| --- | --- | --- |
| Behaviour/Overall Impression | Calm and attentive | 0 |
|  | Lethargic (reduced responsiveness to stimuli) | 1 |
|  | Apathetic (no reaction to external stimuli) | 4 |
| External Appearance (Fur, Eyes) | Normal | 0 |
|  | Slightly dirty/matted (head only) | 1 |
|  | Moderately dirty/matted (body) | 2 |
|  | Very dirty/matted | 3 |
| Nictitating Membrane | Not visible | 0 |
|  | Visible | 3 |
| Food Intake | Normal | 0 |
|  | Reduced | 1 |
|  | Not eating | 2 |
| Water Intake | Normal | 0 |
|  | Reduced | 1 |
|  | Not drinking | 2 |
| Weight Bearing of the operated limb | Normal weight bearing | 0 |
|  | Mild lameness | 1 |
|  | Moderate lameness | 2 |
|  | Severe lameness (no weight bearing) | 3 |
| Paw placement | Normal | 0 |
|  | Knuckling | 1 |
|  | Injury due to permanent knuckling | 3 |
| Coprophagy | Yes | 0 |
|  | No | 1 |
| Rectal Temperature | Normal (38.5 - 39.5 °C) | 0 |
|  | Not within the reference range (too low or too high) | 1 |
| Wound Healing | Normal | 0 |
|  | Signs of inflammation (swelling, redness, etc.) | 2 |
|  | Signs of infection (pus secretion and signs above) | 4 |
| Body Weight | Weight at surgery or 1–5% loss from preoperative weight | 0 |
|  | 6–10% loss from preoperative weight | 1 |
|  | 11–15% loss from preoperative weight | 2 |
|  | >15% loss from preoperative weight | 5 |
| Faecal Consistency | Normal | 0 |
|  | Abnormal | 2 |

Scoring frequency: 2x/day for the first 6 days postop, once on day 7 (day of euthanasia). Weight 1x/day on day 1/3/7.

Abruption criteria: as defined in the score sheet:

Score 3 to 9: Inform veterinarian, diagnostic clarification, possibly shorten observation interval, possibly adjust medication.

Score ≥ 10 for 24 hours despite treatment: Euthanize the individual animal.

Individual score ≥ 5 for 24 hours despite treatment: Euthanize the individual animal.

Day 1-3 postoperatively: Score ≥ 7 for three consecutive days despite treatment: euthanize the individual animal.

Days 4-7 postoperatively: Score ≥ 6 for three consecutive days despite treatment: euthanize the individual animal

Incidents that make the usability of the obtained results for the study impossible: Euthanasia of the individual animal.
