# Supplementary material for: In vivo evaluation of the antibacterial properties of a poly-ε-lysine and hyaluronic acid coated intramedullary implant in a New Zealand White rabbit model
Source: PLoS One. 2026 Mar 4;21(3):e0343597. doi: 10.1371/journal.pone.0343597 (PMC12959695; doi:10.1371/journal.pone.0343597)
Supplement: S2 Table — (DOCX) [file pone.0343597.s005.docx]

**S4 Table. Weight**

| Implant | Rabbit | Weight [kg] | | |
| --- | --- | --- | --- | --- |
|  |  | Day 0 | Day 3 | Day 7 |
| Uncoated | 1 | 3.9 | 3.8 | 3.8 |
|  | 2 | 3.8 | 3.6 | 3.6 |
|  | 3 | 4 | 3.9 | 3.9 |
|  | 4 | 4.3 | 4.2 | 4.1 |
|  | 5 | 3.3 | 3.1 | 3.0 |
|  | 6 | 3.4 | 3.2 | 3.2 |
|  | 7 | 3.4 | 3.5 | 3.4 |
| Coated | 8 | 3.8 | 3.6 | 3.7 |
|  | 9 | 3.9 | 3.7 | 3.6 |
|  | 10 | 3.3 | 3.3 | 3.2 |
|  | 11 | 3.6 | 3.6 | 3.5 |
|  | 12 | 3.2 | 3.0 | 3.1 |
|  | 13 | 3.4 | 3.3 | 3.3 |
|  | 14 | 3.6 | 3.5 | 3.5 |
|  | 15 | 3.3 | 3.2 | 3.2 |
