# Supplementary material for: In vivo evaluation of the antibacterial properties of a poly-ε-lysine and hyaluronic acid coated intramedullary implant in a New Zealand White rabbit model
Source: PLoS One. 2026 Mar 4;21(3):e0343597. doi: 10.1371/journal.pone.0343597 (PMC12959695; doi:10.1371/journal.pone.0343597)
Supplement: S3 Table — Values outside the reference range are in bold (38.5–39.5°C). (DOCX) [file pone.0343597.s006.docx]

**S5 Table. Temperature.** Values outside the reference range are in bold (38.5-39.5°C).

| Implant | Rabbit | Temperature [°C] | | | | | | | | | | | | | | | |
| --- | --- | --- | --- | --- | --- | --- | --- | --- | --- | --- | --- | --- | --- | --- | --- | --- | --- |
|  |  | *Day 0* | *Day 1* | | *Day 2* | | *Day 3* | | *Day 4* | | *Day 5* | | *Day 6* | | | *Day 7* | |
|  |  | *PM* | *AM* | *PM* | *AM* | *PM* | *AM* | *PM* | *AM* | *PM* | *AM* | *PM* | *AM* | *PM* | *AM* | |  |
| Uncoated | 1 | 39.1 | **39.7** | **39.6** | 39.5 | 39.4 | 39.5 | 39.4 | **40.3** | 39.3 | 39.1 | 39.4 | 39.3 | 39.3 | 39.4 | |  |
|  | 2 | 39.1 | 39.3 | 39.4 | **39.6** | 39.4 | 39.5 | 39.2 | **40.1** | 39.5 | 39.2 | 39.2 | **39.6** | 39.4 | 39.3 | |  |
|  | 3 | 39 | 39 | 38.9 | 39.2 | 39.5 | **39.7** | 39 | **41.1** | 39.1 | **39.7** | **39.7** | **40** | **39.8** | **40.1** | |  |
|  | 4 | 39.1 | 39.1 | 38.7 | **39.7** | 38.7 | 39.5 | 38.8 | 39.5 | **39.9** | 39.5 | **39.7** | 39.4 | 39.3 | 39.2 | |  |
|  | 5 | 39.1 | **39.7** | 39.2 | **39.6** | 39.5 | 39.1 | 38.9 | 39.5 | **39.7** | **39.6** | 39.5 | 39.5 | 39.4 | **39.9** | |  |
|  | 6 | 38.6 | 39.4 | 39.3 | 39 | 38.7 | 39.5 | **39.7** | **39.8** | 39.1 | 39.4 | 39.1 | 39 | 38.8 | 39.3 | |  |
|  | 7 | 38.5 | **39.9** | **39.6** | 38.8 | **38.2** | 39.3 | 39.3 | 39.2 | **40.1** | 39.4 | 39.2 | 38.6 | 39 | 39 | |  |
| Coated | 8 | **38.3** | 38.6 | 39.1 | **39.7** | 38.6 | 39.2 | 38.8 | **39.7** | **39.8** | 39.4 | 39.5 | **39.6** | 39.5 | 38.7 | |  |
|  | 9 | 38.9 | 38.7 | 39.3 | 39.1 | 38.9 | 39.2 | **38.4** | 39.2 | **39.6** | 39.2 | 38.5 | 39.4 | 38.5 | 39.2 | |  |
|  | 10 | 38.8 | **39.6** | 39.5 | 39.1 | 38.7 | 39.5 | 39.4 | 39.5 | 39.5 | **39.9** | 38.7 | 39.1 | 38.6 | 39.2 | |  |
|  | 11 | 39 | 38.7 | 39.2 | 39.4 | 39.2 | 39.3 | 39.3 | 39.5 | 39.1 | 39.2 | 39.1 | 39.2 | 38.5 | 39 | |  |
|  | 12 | **38.1** | 39 | 38.9 | 38.9 | 38.8 | **39.6** | 39.2 | 39.3 | 39 | **39.6** | 39.2 | 39.2 | 38.5 | 38.9 | |  |
|  | 13 | 38.5 | 38.7 | 38.6 | **39.6** | 39.2 | 39.5 | 39.3 | 39.2 | 39.2 | 38.7 | 38.9 | 39.1 | 38.8 | 38.7 | |  |
|  | 14 | **37.6** | 39.2 | 38.5 | 39.5 | 39.2 | 39.5 | 38.6 | 39.4 | 39.2 | 39.3 | 38.6 | 38.8 | 38.7 | 39.2 | |  |
|  | 15 | 39.2 | **39.8** | 38.8 | **39.7** | **39.6** | 39.4 | 39.5 | 39.3 | **39.7** | 39.1 | 39.5 | 39.5 | 38.8 | 39.1 | |  |
