# Supplementary material for: In vivo evaluation of the antibacterial properties of a poly-ε-lysine and hyaluronic acid coated intramedullary implant in a New Zealand White rabbit model
Source: PLoS One. 2026 Mar 4;21(3):e0343597. doi: 10.1371/journal.pone.0343597 (PMC12959695; doi:10.1371/journal.pone.0343597)
Supplement: S4 Table — Values outside the reference ranges are in bold: WBC = 5.5–12.5 [*103/µL]; HCT = 33–50 [%]. (DOCX) [file pone.0343597.s007.docx]

**S6 Table. WBC, HCT, CRP.** Values outside the reference ranges are in bold: WBC = 5.5-12.5 [*10^3^/µl]; HCT = 33-50 [%].

| Implant | Rabbit | *Day 0* | | | *Day 3* | | | *Day 7* | | |
| --- | --- | --- | --- | --- | --- | --- | --- | --- | --- | --- |
|  |  | *WBC [10^3^/*µ*l]* | *HCT [%]* | CRP [mg/dl] | *WBC [10^3^/*µ*l]* | *HCT [%]* | CRP [mg/dl] | *WBC [10^3^/*µ*l]* | *HCT [%]* | CRP [mg/dl] |
| Uncoated | 1 | 7.4 | 37 | 0.68 | 9.82 | 37.4 | 0.83 | 9.69 | 36.3 | 0.91 |
|  | 2 | 8.07 | 35.9 | 0.89 | 10.52 | 36 | 0.88 | 12.19 | 33.9 | 0.89 |
|  | 3 | 6.92 | 33.4 | 1.07 | 9.22 | 34.5 | 0.91 | 12.14 | **32.1** | 0.7 |
|  | 4 | 8.53 | 34.9 | 0.99 | 10.61 | 36 | 1 | 7.68 | 35.4 | 1.03 |
|  | 5 | 7.11 | 39.5 | 1.02 | **4.82** | **22.8** | 0.86 | 12.71 | 38.5 | 0.82 |
|  | 6 | 7.85 | 36.9 | 1 | 9.64 | 37.3 | 0.97 | 10.9 | 38 | 0.86 |
|  | 7 | 6.9 | 37.1 | 0.78 | 7.47 | 35.2 | 0.99 | 10.58 | **32.4** | 0.75 |
| Coated | 8 | 6.75 | 34.3 | 0.89 | 10.53 | **32.1** | 0.88 | 8.62 | 34.8 | 0.68 |
|  | 9 | 8.67 | 35.2 | 1.16 | 9.64 | **32** | 1.23 | 11.23 | 34.6 | 0.96 |
|  | 10 | 10.57 | 36.8 | 1.68 | 9.29 | 37.1 | 1.54 | 10.21 | 37.2 | 2.04 |
|  | 11 | 9.59 | 33.9 | 0.85 | 9.44 | 33.8 | 1.08 | 9.53 | 34.2 | 1.14 |
|  | 12 | 8.32 | 36.8 | 1.1 | 12.38 | 37.7 | 1.15 | **14.14** | 36.2 | 0.8 |
|  | 13 | 5.95 | 37 | 1.19 | 8.08 | 37 | 1.22 | 8.19 | 36.3 | 1.21 |
|  | 14 | 5.82 | 36.6 | 1.26 | 6.59 | 36.6 | 1.14 | 8.32 | 34.8 | 1.29 |
|  | 15 | 7 | 37.2 | 1.1 | 10.5 | 37.2 | 1.07 | 11.56 | 35 | 1.1 |
