# Supplementary material for: In vivo evaluation of the antibacterial properties of a poly-ε-lysine and hyaluronic acid coated intramedullary implant in a New Zealand White rabbit model
Source: PLoS One. 2026 Mar 4;21(3):e0343597. doi: 10.1371/journal.pone.0343597 (PMC12959695; doi:10.1371/journal.pone.0343597)
Supplement: S5 Table — (DOCX) [file pone.0343597.s008.docx]

**S7 Table. Protein electrophoresis**

| Implant | Rabbit | Protein content [g/L] | | | | | | | | | | | | | | |
| --- | --- | --- | --- | --- | --- | --- | --- | --- | --- | --- | --- | --- | --- | --- | --- | --- |
|  |  | Dag 0 | | | | | Dag 3 | | | | | Dag 7 | | | | |
|  |  | Total | Albumin | Alpha | Beta | Gamma | Total | Albumin | Alpha | Beta | Gamma | Total | Albumin | Alpha | Beta | Gamma |
| Uncoated | 1 | 56.2 | 38.7 | 5.8 | 10.1 | 1.7 | 70.2 | 42.8 | 8.4 | 16.4 | 2.7 | 65.3 | 42.2 | 6.1 | 14.2 | 2.8 |
|  | 2 | 59.5 | 42.0 | 4.2 | 11.3 | 2.0 | 66.7 | 42.2 | 7.3 | 15.1 | 2.0 | 65.6 | 40.3 | 7.3 | 15.7 | 2.2 |
|  | 3 | 55.7 | 38.6 | 5.4 | 9.1 | 2.6 | 65.8 | 40.5 | 7.0 | 15.3 | 3.0 | 68.4 | 40.3 | 9.2 | 17.2 | 1.6 |
|  | 4 | 63.0 | 39.1 | 7.4 | 12.7 | 3.8 | 67.4 | 40.4 | 7.3 | 16.1 | 3.5 | 73.3 | 45.0 | 8.6 | 15.2 | 4.5 |
|  | 5 | 60.3 | 42.3 | 5.2 | 10.1 | 2.8 | 69.9 | 39.5 | 8.8 | 19.0 | 2.6 | 77.9 | 43.7 | 10.0 | 21.0 | 3.1 |
|  | 6 | 56.3 | 39.5 | 4.1 | 9.1 | 3.7 | 56.6 | 34.2 | 6.4 | 13.0 | 2.9 | 72.4 | 40.8 | 9.2 | 18.9 | 3.5 |
|  | 7 | 56.2 | 36.8 | 5.6 | 11.6 | 2.2 | 64.6 | 37.9 | 8.6 | 16.6 | 1.6 | 69.4 | 47.8 | 7.1 | 11.0 | 3.4 |
| Coated | 8 | 57.3 | 40.2 | 4.6 | 10.7 | 1.8 | 68.5 | 40.8 | 9.0 | 16.3 | 2.4 | 68.3 | 44.9 | 6.7 | 14.2 | 2.5 |
|  | 9 | 55.3 | 39.5 | 4.7 | 8.7 | 2.4 | 63.8 | 39.1 | 7.3 | 14.9 | 2.6 | 68.5 | 42.5 | 7.5 | 15.7 | 2.8 |
|  | 10 | 54.8 | 39.7 | 3.9 | 8.7 | 2.5 | 61.7 | 39.1 | 6.2 | 13.9 | 2.5 | 63.7 | 40.9 | 6.4 | 13.6 | 2.9 |
|  | 11 | 54.9 | 38.6 | 4.2 | 9.3 | 2.7 | 60.8 | 46.0 | 3.8 | 9.3 | 1.8 | 64.7 | 44.3 | 4.9 | 12.4 | 3.1 |
|  | 12 | 56.4 | 41.2 | 3.8 | 8.5 | 2.9 | 68.1 | 43.0 | 6.7 | 14.7 | 3.7 | 60.9 | 40.6 | 5.3 | 13.3 | 1.7 |
|  | 13 | 55.7 | 38.2 | 4.6 | 11.1 | 1.8 | 67.7 | 45.2 | 6.6 | 14.3 | 1.6 | 66.4 | 46.3 | 5.8 | 12.7 | 1.6 |
|  | 14 | 58.8 | 37.6 | 6.6 | 11.9 | 2.7 | Not enough blood sample for analysis | | | | | 64.9 | 38.4 | 7.6 | 15.7 | 3.2 |
|  | 15 | 54.8 | 34.7 | 5.4 | 12.1 | 2.5 | 62.4 | 37.1 | 7.2 | 15.2 | 2.9 | 69.3 | 44.8 | 6.7 | 14.8 | 3.0 |
